# The politics of bodies: gender, coloniality, and prison

**DOI:** 10.11606/s1518-8787.2026060007458

**Published:** 2026-10-26

**Authors:** Carlos Eduardo Rodrigues Toledo

**Affiliations:** I Fundação Oswaldo Cruz Escola Nacional de Saúde Pública Sergio Arouca Rio de Janeiro RJ Brasil Fundação Oswaldo Cruz. Escola Nacional de Saúde Pública Sergio Arouca. Rio de Janeiro, RJ, Brasil

**Keywords:** Prisons, Prisoners, Gender Identity, Public Health, Social Vulnerability

## Abstract

**OBJECTIVE:**

To analyze how gender coloniality and necropolitics materialize in the everyday functioning of the Brazilian prison system, highlighting institutional mechanisms for the production of suffering, disciplining, and slow death, as well as to describe everyday practices of care and solidarity as zones of friction between institutional capture, collective agency, and micropolitical resistances.

**METHODS:**

A qualitative study with a critical-interpretive approach, grounded in critical Public Health, decolonial theory, and feminist critique. It is based on direct professional experience in health care within the prison system of the state of Rio de Janeiro between 2022 and 2024, in facilities housing women, men, and the elderly, within the framework of the Política Nacional de Atenção Integral à Saúde das Pessoas Privadas de Liberdade (National Policy for Comprehensive Health Care for People Deprived of Liberty). The empirical material included field notes, systematized reflective records, and non-traceable analytical narrative reconstructions, organized as institutional vignettes selected for empirical recurrence, ethical relevance, and the ability to express structural mechanisms. The analysis was conducted through successive approaches, combining dense description, identification of institutional patterns, and situated theoretical interpretation.

**RESULTS:**

The analysis was organized around four interconnected themes. The centrality of medicalization in the institutional management of suffering was evident, especially among incarcerated women. Aging in custody was identified as an expression of necropolitics, marked by diagnostic delays, therapeutic discontinuity, and architectural barriers. Systematic barriers to the recognition of trans and transvestite identities were observed, including the refusal to use a social name and the interruption of hormone therapy. Everyday care practices were described as ambiguous zones of friction, at times controlled by authorities and at other times collectively redefined.

**CONCLUSIONS:**

The prison system perpetuates gender coloniality and necropolitics, while care practices create ethical-political fissures that affirm dignity under state custody.

## INTRODUCTION

The Brazilian prison system has never been merely a legal and administrative institution designed to enforce sentences. Rather, it is a historical mechanism for the differential management of life, which perpetuates, in the present, racial, gender, and class hierarchies inherited from the colonial experience. To understand this, we must turn to the concept of the coloniality of power, which describes the persistence of colonial structures in the organization of knowledge, economies, and regimes of recognition of humanity, even after the formal end of colonization^1^. In the prison system, this logic manifests itself in the daily creation of boundaries between lives deemed worthy of care and those treated as disposable^2^.

The structural violence that permeates incarceration in Brazil is not limited to isolated incidents or management failures. In this study, this violence is understood in dialogue with the relationship between violence, social suffering, and public health formulated by Adorno^3^. In this sense, prisons must be understood as a space where vulnerabilities are concentrated, where processes of social exclusion, structural racism, and the precariousness of life converge and intensify^4,5^. Prisons not only reflect preexisting social inequalities but also act as an active mechanism for their reproduction.

Mass incarceration in Brazil thus constitutes a historical continuation of the mechanisms of control and criminalization of racialized populations^6^. Since the era of slavery, Black bodies have been associated with dangerousness, disorder, and social threat, producing a narrative that legitimizes selective criminalization^7^. The transition from the slave-owning regime to peripheral capitalism maintained, in new guises, mechanisms for the criminalization of poverty and the Black body, underpinned by legal, medical, and criminological discourses^9,10^. Contemporary prisons, far from representing a break with this past, perpetuate it in the form of selective incarceration and the punitive management of poverty.

Through the lens of gender coloniality, Western modernity established hierarchical and racialized sexual categories. This was not merely the abstract invention of “man” and “woman,” but the imposition of a normative figure of woman—white, domestic, and submissive—in contrast to the systematic dehumanization of Black and Indigenous women, historically destined for exploitation, hypersexualization, and violence^11^. In contemporary prisons, studies conducted with incarcerated women have related psychotropic medication use to conditions produced by incarceration. These studies identified intense medication use, a tendency to medicalize social issues, and the initiation of psychotropic medication use after imprisonment, associated with prison life, difficulty obtaining medical and psychiatric care, and lack of family contact^12,13^.

Brazilian prisons also function as a prime arena for necropolitics, understood as the exercise of sovereign power to decide who should live and who may be abandoned to a slow death^14^. Unlike classical biopolitics, which focuses on the regulation of life, necropolitics operates through differential exposure to death, whether immediate or prolonged. In everyday prison life, this logic manifests itself in diagnostic delays, therapeutic neglect, inadequate diets, a lack of accessibility for the elderly and the sick, and systematic interruptions in ongoing care. Death, in this context, rarely takes a spectacular form; it is expressed as indefinite waiting, the worsening of preventable illnesses, and the progressive deterioration of bodies.

Colonial hierarchies also permeate dissident identities of gender and sexuality. Incarcerated travestis reported disrespect for their chosen names, denial of hormone treatment, and imprisonment in a men’s prison, conditions that produced identity violations and suffering^15^. The denial of identity recognition and access to specific care constitutes a form of subjective annihilation that compounds the material violence of incarceration. As authors in the feminist and postcolonial fields point out, the subalternized body often expresses itself through symptoms, self-harm, and enforced silences when other forms of speech are prohibited^16,17^.

It is important to clarify that the above formulations regarding the medicalization of women’s suffering, neglect of care in old age, and the erasure of dissident identities are presented here as a synthesis of well-established debates in the literature and as interpretive hypotheses that guide the analysis of the field. At this point in the manuscript, they should not be read as “results” of the study, but rather as analytical categories that will be revisited and elaborated upon based on the institutional vignettes presented below.

Despite its disciplinary power, coloniality does not re-enact itself in the prison without fissures. Throughout every territory of control, micropolitical gaps emerge in which inmates, social networks, and, in certain cases, healthcare workers rearrange the care that is possible. It is important, however, to avoid romanticizing these practices: some of them may be institutionalized (through regulations, programs, volunteer work, or established routines), while others emerge as situated, informal, and contingent forms of agency. In this article, such practices will be treated as zones of friction—sometimes captured by the system, sometimes deviant—in which life asserts itself, while the institution tends to produce waiting, silencing, and slow death.

In the field of Public Health, this reality highlights a central contradiction of the Brazilian state. Although the Unified Health System is based on universality, comprehensiveness, and equity, prison healthcare is affected by structural deficiencies, insufficient coordination between the health and justice sectors, and weaknesses in professional training^18,19^. The presence of healthcare teams in prisons highlights the ongoing tension between a security-first approach and the ethics of care, a tension experienced daily by professionals who must navigate between bureaucratic demands and attending to the needs of people in distress.

Given this scenario, this study adopts a critical perspective grounded in the field of Public Health, decolonial theory, and studies on necropolitics, linked to professional experience in the field of prison health care in the state of Rio de Janeiro. Unlike approaches that treat prisons solely as spaces of rights violations or administrative failure, this study analyzes the prison system as an active mechanism for producing a slow death, in which institutional practices of care, medicalization, neglect of care, and erasure of identity operate as technologies of governance over bodies^14,20,21^.

The objective of this study is to analyze how gender coloniality and necropolitics materialize in the daily life of the Brazilian prison system. By articulating race, gender, aging, dissidence, and institutional care practices, this study understands the prison system simultaneously as an institutional mechanism for the production of slow death and as a space where micropolitical practices emerge that challenge this logic.

## METHODS

This is a qualitative study of a critical-interpretive nature, conducted in the field of Public Health, with systematic engagement with decolonial and anti-colonial frameworks. The methodological design was guided by an understanding of the prison system as an institutional mechanism for the differential management of life and death, in which practices of care, medicalization, and neglect of care function as technologies of governance over bodies in contexts marked by power asymmetries, structural violence, and regimes of exception.

The study was conducted based on the researcher’s professional experience in the field of prison health care in the state of Rio de Janeiro from 2022 to 2024, involving facilities for women, men, and the elderly, within the context of the Política Nacional de Atenção Integral à Saúde das Pessoas Privadas de Liberdade (PNAISP – National Policy for Comprehensive Health Care for Persons Deprived of Liberty). The research explicitly adopts the situated position of the researcher-worker within the Unified Health System, recognizing implication, reflexivity, and narrative responsibility as epistemological conditions for the production of critical knowledge in complex institutional contexts—rather than as biases to be eliminated.

The critical-interpretive analysis was conducted using institutional vignettes constructed from professional records, regulatory documents, and systematized observations in the field of prison health care. These vignettes do not correspond to individual accounts or statistical surveys and were organized in a way that preserves institutional anonymity; they are understood as condensed expressions of structural mechanisms of power, care, and neglect under state custody.

The empirical *corpus* was constituted from field diary notes, systematic reflective records compiled throughout professional practice, and narrative reconstructions of paradigmatic scenes from daily prison life. The scenes were constructed in a non-traceable manner, without any possibility of individual or institutional identification. No medical records, identifiable administrative documents, nominal databases, or any other sources that would allow for the re-identification of incarcerated individuals or healthcare workers were used.

The units of analysis corresponded to institutional vignettes, understood as analytical constructs that condense recurring interactions, flows of care, administrative decisions, and bodily responses to suffering in contexts of incarceration. The vignettes were selected based on three analytical criteria: (i) the recurrence of similar situations observed across different units and time periods; (ii) the ability to express structural mechanisms of power, care, and institutional neglect; and (iii) ethical-political relevance for understanding coloniality, necropolitics, and micropolitical resistances in prison.

The empirical material was organized into four interconnected analytical axes: (1) medicalization and pedagogies of cruelty; (2) aging and necropolitics in prison; (3) dissident identities and institutional erasure; and (4) everyday practices of care and solidarity in prison life, analyzed as zones of friction between institutionalization, collective agency, and micropolitical dynamics of resistance. The analysis was conducted through successive approaches, combining a dense description of the scenes, the identification of recurring institutional mechanisms—such as disciplinary use of medication, neglect of care, erasure of identity, and bureaucratic management of waiting—and theoretical interpretation in dialogue with the theoretical frameworks employed. We sought to ensure interpretive consistency through internal contrast among vignettes, cross-cutting coherence among the analytical axes, and continuous articulation between empirical experience and conceptual construction.

From an ethical standpoint, the study was designed as a critical-interpretive analysis of reflective records and untraceable narrative reconstructions produced within the context of professional practice, without the direct participation of subjects recruited for research, without interviews, without the use of assessment instruments, and without the use of medical records, nominal databases, or identifiable documents. Due to this nature—centered on institutional vignettes that condense patterns of functioning and structural mechanisms—the manuscript was conducted in accordance with the national ethical guidelines applicable to research in the Humanities and Social Sciences and to the protection of individuals in vulnerable contexts, in accordance with Conselho Nacional de Saúde (CNS – National Health Council) Resolutions No. 466/2012 and No. 510/2016. Considering, however, that the prison setting involves power asymmetries and an increased risk of harm, additional safeguards were adopted: removal of names, dates, and facility names; elimination of occupational identifiers; avoidance of identifiable direct speech; and a commitment to risk minimization and institutional non-reidentification, which guided all decisions regarding writing, data selection, and analytical presentation.

Regarding methodological transparency, the empirical data used in this study cannot be shared publicly due to the risk of institutional reidentification and the sensitive nature of the situations analyzed. The vignettes presented constitute untraceable analytical reconstructions, prepared exclusively for the purpose of critical interpretation.

## RESULTS

The results were organized into four interconnected empirical axes, derived from the systematic analysis of institutional vignettes constructed based on the recurrence of situations observed in different prison units and time periods. These axes reflect institutional patterns of suffering management, the organization of care, and everyday responses observed in the prison context: (1) medicalization and institutional management of suffering; (2) aging in incarceration; (3) dissident identities and institutional management of gender; and (4) everyday practices of care and solidarity in the prison system, analyzed as zones of friction between institutionalization, collective agency, and micropolitical dynamics of resistance.

### Medicalization and Institutional Management of Suffering

The case studies analyzed showed that the institutional management of suffering in prison occurred predominantly through medicalizing strategies, with a particular prevalence among incarcerated women. Frequent and repeated prescriptions of anxiolytics and antipsychotics were observed, often without systematic individualized clinical evaluation or continuous longitudinal follow-up. Complaints related to insomnia, anxiety, persistent sadness, crying spells, and emotional distress were repeatedly recorded as nonspecific psychiatric diagnoses, such as anxiety disorders or sleep disorders.

Situations associated with a history of domestic violence, forced separation from children, grief, and material hardship were predominantly addressed by expanding pharmacological regimens, with limited access to qualified counseling or structured psychosocial support. In various facilities, prescriptions were maintained for prolonged periods, even in the face of repeated reports of adverse effects, such as excessive sedation, mental confusion, and difficulty concentrating.

Among elderly men in custody, the management of suffering took a distinct form. The case studies revealed repeated delays in diagnoses, successive postponements of tests and surgical procedures, interruptions in ongoing treatments, and shortages of medications for continuous use. Requests for care went unanswered for prolonged periods, and recurring statements expressed a perception of continuous waiting and the progressive worsening of health conditions.

Among transgender and transvestite individuals deprived of liberty, acute manifestations of distress—including emotional crises, self-harm, and suicide attempts—were frequently followed by an increase in medication prescriptions, with no record of ongoing psychosocial follow-up or specific care strategies tailored to the needs of this population.

### Aging in Detention

Aging in prison was identified in the case studies as a phenomenon observed repeatedly across different facilities. There was an increase in the number of older adults deprived of their liberty, without corresponding changes in the organization of physical spaces or care pathways. The predominant profile observed consisted of older men with a history of poverty, low educational attainment, and past experiences of social exclusion.

The physical conditions of the facilities presented limitations in meeting the needs of this population. Overcrowding, a lack of ramps, the absence of accessible restrooms, and restricted mobility for people with motor impairments were observed. Older adults with mobility difficulties often depended on other inmates for feeding, personal hygiene, and moving around within the facilities.

Access to health care was irregular. Chronic conditions such as hypertension, diabetes mellitus, and heart disease were identified but lacked continuous follow-up. Referrals for specialized consultations and diagnostic tests were postponed or canceled, often due to the lack of an escort, and medications for ongoing treatment arrived at the facilities only intermittently.

Despite these limitations, informal care strategies were observed among the inmates themselves. In different units, initiatives of mutual support were documented to remind others of medication schedules, assist peers with neurological sequelae, and notify healthcare professionals when signs of clinical deterioration arose, constituting collective responses to the gaps in care observed in daily institutional life.

### Dissident Identities and Institutional Management of Gender

The case studies analyzed revealed systematic barriers to the institutional recognition of the gender identities of transgender and transvestite individuals deprived of their liberty. We observed refusals to use social names, interruptions in or lack of regular access to hormone therapy, and compulsory placement in wards incompatible with gender identity.

These practices were associated with recurrent physical and emotional distress. The interruption of hormone therapy was accompanied by physical symptoms and an exacerbation of psychological distress. The compulsory use of the legal name was described as a repeated experience of institutional humiliation. Even when placed in specific wings, some individuals remained in a state of isolation, with restricted access to group activities and services.

Episodes of verbal and physical violence were also reported, perpetrated by both correctional officers and other inmates. In contrast, isolated instances of recognition of the social name by fellow inmates and healthcare professionals were observed, described as isolated gestures of respect and recognition within a predominantly hostile context.

### Everyday Care Practices in the Prison System

Alongside situations of violence and the lack of an institutional response, everyday care practices emerged, developed by inmates and, in some contexts, by healthcare professionals. These practices were not treated as external to the prison system but as rearrangements produced within the institutions, sometimes guided by formal routines, sometimes by informal and contingent routines, creating zones of friction between institutional confinement and collective agency.

In women’s units, therapeutic workshops on embroidery, painting, and writing were documented as spaces for social interaction and the sharing of experiences. When supported by institutional staff, these activities functioned as a formal provision of care; when appropriated and reinterpreted by the female inmates, they served as strategies for mutual support and the rebuilding of bonds in settings marked by social and emotional rupture.

Memory groups and conversation circles, organized in both men’s and women’s units, served as spaces for exchange and recognition. In clinical care, low-visibility practices—such as addressing inmates by name, adjusting treatment regimens to the specific conditions of the prison, and maintaining a minimum level of continuity in follow-up—were documented as everyday strategies capable of producing clinical and symbolic differences in care.

In the context of Directly Observed Treatment for tuberculosis, informal support networks were observed among female inmates aimed at reminding each other of medication schedules, monitoring adverse effects, and encouraging adherence to treatment. Spirituality was present through the organization of singing circles, prayers, and collective rituals, oscillating between a potentially pacifying dimension—when strictly supervised—and a dimension of symbolic reappropriation of the body and time—when functioning as a repertoire of belonging and mutual support.

Practices of material solidarity, such as the sharing of food, clothing, hygiene products, and medications, were observed among inmates without regular access to basic necessities, forming a moral economy of care that responds, in a context-specific way, to the immediate needs arising from the structural scarcity within the prison.

## DISCUSSION

The results of this study show that the Brazilian prison system functions as a privileged space for the contemporary manifestation of gender coloniality and necropolitics, through institutional mechanisms that produce suffering, discipline, and a slow death. The systematic medicalization of incarcerated women, the failure to provide care to the elderly, the institutional erasure of dissident identities, and the predominant racial and social profile of the incarcerated population do not constitute isolated management failures, but rather coordinated expressions of a structural regime of governance over bodies, which differentially administers life and death^1,14,22^.

The relationship between violence, social suffering, and public health provides a conceptual basis for interpreting the effects of the conditions described in this study^3^. In the prison context, these effects are associated with the rights violations and degrading conditions discussed in the literature^5^. The findings of this study reinforce this interpretation by showing that seemingly routine practices—such as the repeated prescription of psychotropic drugs, the postponement of medical appointments, and the denial of identity recognition—function as regular technologies of governance, integrated into the daily operations of prison institutions.

The medicalization of women’s suffering observed in this study aligns with research conducted in Brazilian women’s prisons. In one prison in Rio Grande do Sul, researchers identified intense psychotropic medication use, insufficient comprehensive care, and a tendency to medicalize social issues as a form of control and containment^12^. In another women’s prison, 58.8% of the women who used psychotropic medication began using it after imprisonment; prison life, difficulty obtaining medical and psychiatric care, and lack of family contact were identified as related factors^13^. In the present study, repeated prescriptions and insufficient opportunities for listening were identified in the cases analyzed and interpreted in light of this evidence.

Regarding aging during incarceration, the results align with Brazilian studies indicating growth in the older incarcerated population and the inadequacy of prisons in meeting this population’s healthcare needs^23^. Among incarcerated older adults, aging has also been associated with losses, limitations, and suffering intensified by the conditions of confinement^24^. In the present study, prolonged waiting, interruption of treatment, and lack of accessibility were identified in the cases analyzed and interpreted as manifestations of neglect of care.

The social profile observed among people deprived of liberty confirms the structural role of mass incarceration as a cog in the machinery of institutional racism. National data indicate that the Brazilian criminal justice system acts as a primary mechanism for managing poverty and the Black population, exacerbating pre-existing inequalities in access to education, employment, and health care^4,10,25^.

Regarding gender-diverse identities, the findings align with studies on the violations experienced by travestis and transgender women in the prison system. Disrespect for chosen names, denial of hormone treatment, and imprisonment in men’s institutions compromise identity recognition and access to care^15^. Narratives of incarcerated travestis and transgender women also show institutional difficulties in responding to transfeminine bodies and identities, as well as continuing violations during imprisonment^26^.

The tension between the logic of security and the ethics of care emerges in this study as a central axis of the institutional practices analyzed. The PNAISP establishes guidelines for comprehensiveness, intersectoral coordination, decentralization, hierarchical organization, and humanization^27^. At the institutional level, the execution of penal policies involves challenges in management, coordination and implementation^28^. However, a study conducted in prisons in Paraíba identified structural deficiencies, insufficient coordination between the health and justice sectors, and weaknesses in professional training^19^.

At the same time, the results highlight the presence of everyday practices of care and solidarity that challenge, without romanticizing them, the dominant colonial and necropolitical logic. Workshops, memory groups, informal treatment support networks, practices of material solidarity, and expressions of spirituality should not be automatically understood as insurgency, but rather as zones of friction in which institutional practices, collective appropriations, and micropolitical dynamics of resistance intertwine^20,21,29,30^.

From a theoretical-analytical perspective, the findings confirm the relevance of articulating gender coloniality, necropolitics, and the micropolitics of care to understand the functioning of the Brazilian prison system. Medicalization, the denial of care, and the erasure of identity constitute structural components of a regime of governance that selects which lives should be protected and which can be progressively eliminated^1,2,14,22^. At the same time, the micropolitical gaps and everyday rearrangements of care observed indicate that life is never completely captured by colonial logic, giving rise to bonds, narratives, and alternative ways of existing^29^.

For the field of Public Health, this study indicates the need to deepen the critical analysis of institutional violence and neglect of care in the prison system. At the same time, Permanent Health Education experiences in this context show that discussion groups can promote in-depth discussions, collective constructions, and proposals for intervention^31^. Based on the findings of the present study, these care practices are interpreted as an ethical and political dimension in confronting necropolitics^14,32,33^.

## FINAL CONSIDERATIONS

This study analyzed the Brazilian prison system as a privileged territory for the contemporary manifestation of gender coloniality and necropolitics, highlighting how institutional practices of medicalization, neglect of care, erasure of identity, and racial selectivity operate in an interconnected manner to produce lives considered disposable. The results demonstrate that prison is not limited to the enforcement of sentences but acts as an active device for the governance of bodies, in which the management of pain, waiting, and invisibility constitutes a central technology of punishment.

At the same time, the study revealed that, even in contexts of extreme institutional violence, institutional and collective care practices emerge that challenge the dominant colonial logic. Therapeutic workshops, memory groups, solidarity networks supporting treatment, spiritual practices, and everyday gestures of recognition constitute concrete responses to the state’s absence, operating as zones of friction within the necropolitical apparatus and affirming dignity where slow death tends to be normalized. These practices should not be romanticized as external to the system: they vary between institutionalized offerings and situated collective appropriations but nonetheless constitute recurring repertoires for the production of care and meaning within the prison.

In the field of Public Health, there is a need to reposition prison health as a strategic dimension in addressing the structural inequalities that permeate the Brazilian penal system.

Prison perpetuates gender coloniality and necropolitics, while care practices create ethical-political fissures that affirm dignity under state custody.

Although the PNAISP represents a significant normative advance, its implementation remains insufficient to halt the mechanisms of care neglect, excessive medicalization, and erasure of identity observed in this study. Guaranteeing the right to health in this context requires not only expanding care coverage but also reorienting the care model by incorporating practices based on listening, bonding, recognition, and shared responsibility.

From an ethical-political perspective, rethinking care in prisons implies questioning the centrality of mass incarceration as a policy for managing poverty, race, gender, and dissidence. In a society marked by a legacy of slavery and structural racism, the normalization of the slow death of women, older adults, transgender people, and Black youth reveals the persistence of coloniality as the organizing principle of policies of punishment and social control.

Care, when practiced as skilled listening, bonding, and recognition, constitutes a concrete strategy for confronting necropolitics in everyday prison life. Beyond the alleviation of individual suffering, these practices re-inscribe bodies within the realm of humanity and generate possible alternatives to the dominant punitive logic. Recognizing, strengthening, and institutionalizing such experiences is a central task for Public Health committed to human dignity, social justice, and the critical transformation of contemporary regimes of punishment.
