# Supplementary material for: The politics of bodies: gender, coloniality, and prison
Source: Rev Saude Publica. 2026 Oct 26;60:e48. doi: 10.11606/s1518-8787.2026060007458 (PMC13753556; doi:10.11606/s1518-8787.2026060007458)
Supplement: Translation

# Translation: Política dos corpos: gênero, colonialidade e prisão

**Authors:** Carlos Eduardo Rodrigues Toledo

**Affiliations:** I Brasil Fundação Oswaldo Cruz. Escola Nacional de Saúde Pública Sergio Arouca. Rio de Janeiro, RJ, Brasil

**Keywords:** Prisões, Prisioneiros, Identidade de Gênero, Saúde Pública, Vulnerabilidade Social

## Abstract

**OBJETIVO:**

Analisar como a colonialidade de gênero e a necropolítica se materializam no cotidiano do sistema prisional brasileiro, evidenciando dispositivos institucionais de produção de sofrimento, disciplinamento e morte lenta, bem como descrever práticas cotidianas de cuidado e solidariedade como zonas de fricção entre captura institucional, agência coletiva e resistências micropolíticas.

**MÉTODOS:**

Estudo qualitativo de abordagem crítico-interpretativa, ancorado na Saúde Coletiva crítica, na teoria decolonial e na crítica feminista. Baseia-se em experiência profissional direta no cuidado em saúde no sistema prisional do estado do Rio de Janeiro, entre 2022 e 2024, em unidades femininas, masculinas e com população idosa, no âmbito da Política Nacional de Atenção Integral à Saúde das Pessoas Privadas de Liberdade. O material empírico incluiu diário de campo, registros reflexivos sistematizados e reconstruções narrativas analíticas não rastreáveis, organizadas como vinhetas institucionais selecionadas por recorrência empírica, relevância ética e capacidade de expressar mecanismos estruturais. A análise foi conduzida por aproximações sucessivas, articulando descrição densa, identificação de padrões institucionais e interpretação teórica situada.

**RESULTADOS:**

A análise organizou-se em quatro eixos interligados. Evidenciou-se a centralidade da medicalização no manejo institucional do sofrimento, especialmente entre mulheres privadas de liberdade. Identificou-se o envelhecimento sob custódia como expressão de necropolítica, marcado por atrasos diagnósticos, descontinuidade terapêutica e barreiras arquitetônicas. Observaram-se barreiras sistemáticas ao reconhecimento de identidades trans e travestis, incluindo recusa do nome social e interrupção da hormonioterapia. Descreveram-se práticas cotidianas de cuidado como zonas ambíguas de fricção, ora tuteladas, ora ressignificadas coletivamente.

**CONCLUSÕES:**

O cárcere atualiza a colonialidade de gênero e a necropolítica, enquanto práticas de cuidado produzem fissuras ético-políticas que afirmam dignidade sob custódia estatal.

## INTRODUÇÃO

O cárcere brasileiro nunca foi apenas uma instituição jurídico-administrativa destinada à execução da pena. Trata-se, antes, de uma engrenagem histórica de gestão diferencial da vida, que atualiza, no presente, hierarquias raciais, de gênero e de classe herdadas da experiência colonial. Para compreendê-lo, é necessário recorrer ao conceito de colonialidade do poder, que descreve a persistência de estruturas coloniais na organização dos saberes, das economias e dos regimes de reconhecimento da humanidade, mesmo após o fim formal da colonização^1^. No sistema prisional, essa lógica se materializa na produção cotidiana de fronteiras entre vidas consideradas dignas de cuidado e existências tratadas como descartáveis^2^.

A violência estrutural que atravessa o encarceramento no Brasil não se limita a episódios pontuais ou a falhas de gestão. Neste estudo, essa violência é compreendida em diálogo com a relação entre violência, sofrimento social e saúde pública formulada por Adorno^3^. Nesse sentido, o cárcere deve ser compreendido como espaço privilegiado de concentração de vulnerabilidades, onde processos de exclusão social, racismo estrutural e precarização da vida se condensam e se intensificam^4,5^. A prisão não apenas reflete desigualdades sociais preexistentes, mas atua como dispositivo ativo de sua reprodução.

O encarceramento em massa no Brasil constitui, assim, uma continuidade histórica dos mecanismos de controle e criminalização de populações racializadas^6^. Desde o período escravocrata, corpos negros foram associados à periculosidade, à desordem e à ameaça social, produzindo uma narrativa que legitima a seletividade penal^7^. A transição do regime escravocrata para o capitalismo periférico manteve, sob novas roupagens, dispositivos de criminalização da pobreza e do corpo negro, sustentados por discursos jurídicos, médicos e criminológicos^9,10^. As prisões contemporâneas, longe de representarem ruptura com esse passado, atualizam-no sob a forma de encarceramento seletivo e gestão punitiva da miséria.

Na chave da colonialidade de gênero, a modernidade ocidental instituiu categorias sexuais hierarquizadas e racializadas. Não se tratou apenas da invenção abstrata de “homem” e “mulher”, mas da imposição de uma figura normativa de mulher – branca, doméstica e submissa – em contraste com a desumanização sistemática de mulheres negras e indígenas, historicamente destinadas à exploração, à hipersexualização e à violência^11^. No cárcere contemporâneo, estudos realizados com mulheres privadas de liberdade relacionam o uso de psicofármacos às condições produzidas pelo encarceramento. Foram identificados uso intenso desses medicamentos, tendência à medicalização de questões sociais e início do consumo após a prisão, associado ao cotidiano prisional, à dificuldade de assistência médico-psiquiátrica e à ausência de contato familiar^12,13^.

A prisão brasileira também se configura como território privilegiado da necropolítica, entendida como o exercício do poder soberano de decidir quem deve viver e quem pode ser abandonado à morte lenta^14^. Diferentemente da biopolítica clássica, centrada na regulação da vida, a necropolítica opera por meio da exposição diferencial à morte, seja ela imediata ou prolongada. No cotidiano prisional, essa lógica se manifesta em atrasos diagnósticos, negligência terapêutica, dietas inadequadas, ausência de acessibilidade para pessoas idosas e adoecidas e interrupções sistemáticas de cuidados continuados. A morte, nesse contexto, raramente assume forma espetacular; ela se expressa como espera indefinida, agravamento de doenças evitáveis e deterioração progressiva dos corpos.

As hierarquias coloniais atravessam, ainda, as identidades dissidentes de gênero e sexualidade. Travestis privadas de liberdade relataram desrespeito ao nome social, impedimento da hormonização e encarceramento em uma penitenciária masculina, condições que produziram violações de identidade e sofrimento^15^. A negação do reconhecimento identitário e do acesso a cuidados específicos configura uma forma de aniquilação subjetiva que se soma às violências materiais do encarceramento. Como apontam autoras do campo feminista e pós-colonial, o corpo subalternizado frequentemente se expressa por meio de sintomas, automutilações e silêncios forçados, quando outras formas de fala são interditadas^16,17^.

Importa delimitar que as formulações acima sobre medicalização do sofrimento feminino, abandono assistencial no envelhecer e apagamento de identidades dissidentes são aqui mobilizadas como síntese de debates consolidados na literatura e como hipóteses interpretativas que orientam a leitura do campo. Elas não devem ser lidas, neste ponto do manuscrito, como “resultados” do estudo, mas como categorias analíticas que serão retomadas e densificadas a partir das vinhetas institucionais apresentadas adiante.

Apesar de sua potência disciplinar, a colonialidade não se reinscreve no cárcere sem fissuras. Em todo território de controle emergem brechas micropolíticas nas quais internos, redes de convivência e, em certos casos, trabalhadores da saúde rearranjam o cuidado possível. Importa, contudo, evitar romantizações: parte dessas práticas pode estar institucionalizada (por normas, programas, voluntariado ou rotinas previstas) e parte emerge como agência situada, informal e contingente. Neste artigo, tais práticas serão tratadas como zonas de fricção – ora capturadas pelo dispositivo, ora desviantes – nas quais se afirma a vida, enquanto a instituição tende a produzir espera, silenciamento e morte lenta.

No campo da saúde coletiva, essa realidade explicita uma contradição central do Estado brasileiro. Embora o Sistema Único de Saúde se fundamente na universalidade, integralidade e equidade, a atenção à saúde nas prisões apresenta carências estruturais, desarticulação entre os setores da saúde e da justiça e fragilidades na formação dos profissionais^18,19^. A presença de equipes de saúde nas unidades prisionais revela a tensão permanente entre a lógica securitária e a ética do cuidado, vivida cotidianamente por profissionais que transitam entre exigências burocráticas e a escuta de corpos em sofrimento.

Diante desse cenário, este estudo adota uma perspectiva crítica ancorada no campo da saúde coletiva, na teoria decolonial e nos estudos sobre necropolítica, articulada à experiência profissional no campo da saúde prisional no estado do Rio de Janeiro. Diferentemente de abordagens que tratam o cárcere apenas como espaço de violação de direitos ou falha administrativa, este estudo analisa o sistema prisional como dispositivo ativo de produção da morte lenta, no qual práticas institucionais de cuidado, medicalização, abandono assistencial e apagamento identitário operam como tecnologias de governo dos corpos^14,20,21^.

O objetivo deste estudo é analisar de que modo a colonialidade de gênero e a necropolítica se materializam no cotidiano do sistema prisional brasileiro. Ao articular raça, gênero, envelhecimento, dissidências e práticas institucionais de cuidado, este estudo compreende o sistema prisional simultaneamente como dispositivo institucional de produção da morte lenta e como espaço de emergência de práticas micropolíticas que tensionam essa lógica.

## MÉTODOS

Trata-se de um estudo qualitativo, de natureza crítico-interpretativa, desenvolvido no campo da saúde coletiva, com diálogo sistemático com referenciais decoloniais e anticoloniais. O desenho metodológico foi orientado pela compreensão do sistema prisional como dispositivo institucional de gestão diferencial da vida e da morte, no qual práticas de cuidado, medicalização e abandono assistencial operam como tecnologias de governo dos corpos em contextos marcados por assimetrias de poder, violência estrutural e regimes de exceção.

O estudo foi conduzido a partir da experiência profissional do pesquisador no âmbito da saúde prisional, no estado do Rio de Janeiro, no período de 2022 a 2024, envolvendo unidades femininas, masculinas e com população idosa, no contexto da Política Nacional de Atenção Integral à Saúde das Pessoas Privadas de Liberdade (PNAISP). A investigação assume explicitamente a posição situada do pesquisador-trabalhador do Sistema Único de Saúde, reconhecendo a implicação, a reflexividade e a responsabilidade narrativa como condições epistemológicas da produção de conhecimento crítico em contextos institucionais complexos, e não como vieses a serem eliminados.

A análise crítico-interpretativa foi realizada a partir de vinhetas institucionais construídas com base em registros profissionais, documentos normativos e observações sistematizadas no campo da saúde prisional. Essas vinhetas não correspondem a relatos individuais nem a levantamento estatístico e foram organizadas de modo a preservar o anonimato institucional, sendo compreendidas como expressões condensadas de mecanismos estruturais de poder, cuidado e abandono sob custódia do Estado.

O *corpus* empírico foi constituído a partir de anotações em diário de campo, registros reflexivos sistematizados ao longo da prática profissional e reconstruções narrativas de cenas paradigmáticas do cotidiano prisional. As cenas foram elaboradas de modo não rastreável, sem qualquer possibilidade de identificação individual ou institucional. Não foram utilizados prontuários clínicos, documentos administrativos identificáveis, bases de dados nominais ou quaisquer outras fontes que permitissem a reidentificação de pessoas privadas de liberdade ou de trabalhadores da saúde.

As unidades de análise corresponderam a vinhetas institucionais, compreendidas como construções analíticas que condensam interações recorrentes, fluxos de cuidado, decisões administrativas e respostas corporais ao sofrimento em contextos de privação de liberdade. As vinhetas foram selecionadas a partir de três critérios analíticos: (i) recorrência de situações semelhantes observadas em diferentes unidades e períodos; (ii) capacidade de expressar mecanismos estruturais de poder, cuidado e abandono institucional; e (iii) relevância ético-política para a compreensão da colonialidade, da necropolítica e das resistências micropolíticas no cárcere.

O material empírico foi organizado em quatro eixos analíticos interligados: (1) medicalização e pedagogias da crueldade; (2) envelhecimento e necropolítica no cárcere; (3) identidades dissidentes e apagamento institucional; e (4) práticas cotidianas de cuidado e solidariedade no cotidiano prisional, analisadas como zonas de fricção entre institucionalização, agência coletiva e dinâmicas micropolíticas de resistência. A análise foi conduzida por aproximações sucessivas, articulando descrição densa das cenas, identificação de mecanismos institucionais recorrentes – como disciplinamento medicamentoso, omissão assistencial, apagamento identitário e gestão burocrática da espera – e interpretação teórica em diálogo com os referenciais mobilizados. Buscou-se assegurar consistência interpretativa por meio do contraste interno entre vinhetas, da coerência transversal entre os eixos analíticos e da articulação contínua entre experiência empírica e construção conceitual.

Do ponto de vista ético, o estudo foi construído como análise crítico-interpretativa de registros reflexivos e reconstruções narrativas não rastreáveis, produzidas no âmbito da prática profissional, sem participação direta de sujeitos recrutados para pesquisa, sem entrevistas, sem aplicação de instrumentos, sem uso de prontuários, bases nominais ou documentos identificáveis. Por essa natureza – centrada em vinhetas institucionais que condensam padrões de funcionamento e mecanismos estruturais – o manuscrito foi conduzido sob os referenciais éticos nacionais aplicáveis às pesquisas em Ciências Humanas e Sociais e à proteção de pessoas em contextos de vulnerabilidade, conforme as Resoluções do Conselho Nacional de Saúde (CNS) nº 466/2012 e nº 510/2016. Considerando, entretanto, que o campo prisional envolve assimetrias de poder e risco ampliado de dano, adotaram-se salvaguardas adicionais: supressão de nomes, datas e unidades; eliminação de marcadores ocupacionais; não utilização de falas diretas identificáveis; e compromisso de minimização de risco e não reidentificação institucional, orientando todas as escolhas de escrita, recorte e exposição analítica.

Quanto à transparência metodológica, os dados empíricos utilizados neste estudo não são passíveis de compartilhamento público, em razão do risco de reidentificação institucional e da natureza sensível das situações analisadas. As vinhetas apresentadas constituem reconstruções analíticas não rastreáveis, elaboradas exclusivamente para fins de interpretação crítica.

## RESULTADOS

Os resultados foram organizados em quatro eixos empíricos interligados, derivados da análise sistemática de vinhetas institucionais construídas a partir da recorrência de situações observadas em diferentes unidades prisionais e períodos. Esses eixos expressam padrões institucionais de manejo do sofrimento, organização do cuidado e respostas cotidianas observadas no contexto prisional: (1) medicalização e manejo institucional do sofrimento; (2) envelhecimento em privação de liberdade; (3) identidades dissidentes e gestão institucional do gênero; e (4) práticas cotidianas de cuidado e solidariedade no sistema prisional, analisadas como zonas de fricção entre institucionalização, agência coletiva e dinâmicas micropolíticas de resistência.

### Medicalização e Manejo Institucional do Sofrimento

As vinhetas analisadas evidenciaram que o manejo institucional do sofrimento no cárcere ocorreu predominantemente por meio de estratégias medicalizantes, com especial incidência entre mulheres privadas de liberdade. Observou-se prescrição frequente e reiterada de ansiolíticos e antipsicóticos, muitas vezes sem avaliação clínica individualizada sistemática ou acompanhamento longitudinal continuado. Queixas relacionadas à insônia, à ansiedade, à tristeza persistente, a crises de choro e ao sofrimento emocional foram, de modo recorrente, registradas como diagnósticos psiquiátricos inespecíficos, como transtornos de ansiedade ou distúrbios do sono.

Situações associadas a histórico de violência doméstica, separação compulsória de filhos, luto e precariedade material foram respondidas predominantemente por ampliação de esquemas farmacológicos, com restrita oferta de escuta qualificada ou acompanhamento psicossocial estruturado. Em diferentes unidades, registrou-se a manutenção prolongada de prescrições, mesmo diante de relatos reiterados de efeitos adversos, como sedação excessiva, confusão mental e dificuldade de concentração.

Entre homens idosos privados de liberdade, o manejo do sofrimento apresentou configuração distinta. As vinhetas evidenciaram atrasos reiterados em diagnósticos, adiamentos sucessivos de exames e procedimentos cirúrgicos, interrupções de tratamentos em curso e desabastecimento de medicamentos de uso contínuo. Demandas por cuidado permaneceram sem resposta por períodos prolongados, e falas recorrentes expressaram a percepção de espera contínua e agravamento progressivo das condições de saúde.

Entre pessoas trans e travestis privadas de liberdade, manifestações agudas de sofrimento – incluindo crises emocionais, automutilações e tentativas de suicídio – foram frequentemente seguidas por intensificação da prescrição medicamentosa, sem registro de acompanhamento psicossocial contínuo ou de estratégias de cuidado específicas voltadas às necessidades dessa população.

### Envelhecimento em Privação de Liberdade

O envelhecimento no cárcere foi identificado nas vinhetas como um fenômeno observado de forma recorrente em diferentes unidades. Registrou-se aumento do número de pessoas idosas privadas de liberdade, sem alterações correspondentes na organização dos espaços físicos ou dos fluxos assistenciais. O perfil predominante observado foi composto por homens com idade avançada, histórico de pobreza, baixa escolaridade e trajetórias anteriores de exclusão social.

As condições físicas das unidades apresentaram limitações para atender às necessidades dessa população. Foram observadas superlotação, inexistência de rampas, ausência de sanitários adaptados e circulação restrita para pessoas com limitações motoras. Idosos com dificuldades de locomoção dependeram frequentemente de outros internos para alimentação, higiene pessoal e deslocamento dentro das unidades.

O acesso à atenção à saúde ocorreu de forma irregular. Doenças crônicas como hipertensão arterial, diabetes mellitus e cardiopatias foram identificadas sem acompanhamento contínuo. Encaminhamentos para consultas especializadas e exames complementares foram adiados ou cancelados, frequentemente por ausência de escolta, e medicamentos de uso contínuo chegaram de forma intermitente às unidades.

Apesar dessas limitações, observaram-se estratégias informais de cuidado entre os próprios internos. Em diferentes unidades, foram registradas iniciativas de apoio mútuo para lembrar horários de medicação, auxiliar colegas com sequelas neurológicas e comunicar profissionais de saúde diante de sinais de agravamento clínico, configurando respostas coletivas às lacunas assistenciais observadas no cotidiano institucional.

### Identidades Dissidentes e Gestão Institucional do Gênero

As vinhetas analisadas evidenciaram barreiras sistemáticas ao reconhecimento institucional das identidades de gênero de pessoas trans e travestis privadas de liberdade. Foram observadas recusa no uso do nome social, interrupção ou inexistência de acesso regular à hormonioterapia e alocação compulsória em alas incompatíveis com a identidade de gênero.

Essas práticas estiveram associadas ao sofrimento físico e emocional recorrente. A interrupção da hormonioterapia foi acompanhada de sintomas físicos e agravamento do sofrimento psíquico. O uso compulsório do nome de registro foi descrito como experiência reiterada de constrangimento institucional. Mesmo quando alocadas em alas específicas, algumas pessoas permaneceram em situação de isolamento, com restrição de acesso a atividades coletivas e serviços.

Também foram registrados episódios de violência verbal e física, praticados tanto por agentes penitenciários quanto por outros internos. Em contraponto, observaram-se episódios pontuais de reconhecimento do nome social por parte de colegas e profissionais de saúde, descritos como gestos isolados de respeito e reconhecimento no interior de um contexto predominantemente hostil.

### Práticas Cotidianas de Cuidado no Sistema Prisional

Ao lado das situações de violência e ausência de resposta institucional, emergiram práticas cotidianas de cuidado desenvolvidas por internos e, em alguns contextos, por profissionais de saúde. Essas práticas não foram tratadas como externas ao dispositivo prisional, mas como rearranjos produzidos no interior das instituições, ora previstos por rotinas formais, ora informais e contingentes, configurando zonas de fricção entre captura institucional e agência coletiva.

Em unidades femininas, oficinas terapêuticas de bordado, pintura e escrita foram registradas como espaços de convivência e partilha de experiências. Quando sustentadas por equipes institucionais, essas atividades operaram como oferta formal de cuidado; quando apropriadas e ressignificadas pelas internas, funcionaram como estratégias de apoio mútuo e reconstrução de vínculos em cenários marcados pela ruptura social e afetiva.

Grupos de memória e rodas de conversa, organizados em unidades masculinas e femininas, constituíram espaços de troca e reconhecimento. No acompanhamento clínico, práticas de baixa visibilidade – como chamar internos pelo nome, ajustar esquemas terapêuticos às condições concretas do cárcere e manter continuidade mínima do seguimento – foram registradas como estratégias cotidianas capazes de produzir diferenças clínicas e simbólicas no cuidado.

No contexto do Tratamento Diretamente Observado da tuberculose, observaram-se redes informais de apoio entre internas voltadas a lembrar horários de medicação, monitorar efeitos adversos e estimular a adesão ao tratamento. A espiritualidade esteve presente por meio da organização de rodas de canto, orações e rituais coletivos, oscilando entre uma dimensão potencialmente pacificadora, quando rigidamente tutelada, e uma dimensão de reapropriação simbólica do corpo e do tempo, quando operada como repertório de pertencimento e suporte mútuo.

Práticas de solidariedade material, como o compartilhamento de alimentos, roupas, produtos de higiene e medicamentos, foram registradas entre internos sem acesso regular a bens básicos, compondo uma economia moral do cuidado que responde, de forma situada, às necessidades imediatas produzidas pela escassez estrutural do cárcere.

## DISCUSSÃO

Os resultados deste estudo evidenciam que o sistema prisional brasileiro opera como território privilegiado de atualização contemporânea da colonialidade de gênero e da necropolítica, por meio de dispositivos institucionais que produzem sofrimento, disciplinamento e morte lenta. A medicalização sistemática de mulheres privadas de liberdade, a omissão assistencial dirigida a pessoas idosas, o apagamento institucional de identidades dissidentes e o perfil racial e social predominante da população encarcerada não se configuram como falhas pontuais de gestão, mas como expressões articuladas de um regime estrutural de governo dos corpos, que administra diferencialmente a vida e a morte^1,14,22^.

A relação entre violência, sofrimento social e saúde pública fornece uma base conceitual para interpretar os efeitos das condições descritas neste estudo^3^. No contexto prisional, esses efeitos aparecem associados às violações de direitos e às condições degradantes discutidas pela literatura^5^. Os achados deste estudo reforçam essa interpretação ao evidenciar que práticas aparentemente rotineiras – como prescrição reiterada de psicofármacos, adiamento de atendimentos e negação de reconhecimento identitário – operam como tecnologias regulares de governo, integradas ao funcionamento cotidiano das instituições prisionais.

A medicalização do sofrimento feminino observada neste estudo dialoga com pesquisas realizadas em unidades prisionais femininas brasileiras. Em uma unidade do Rio Grande do Sul, foram identificados uso intenso de psicofármacos, insuficiência do cuidado integral e tendência à medicalização de questões sociais como forma de controle e contenção^12^. Em outra prisão feminina, 58,8% das mulheres que utilizavam psicotrópicos começaram o consumo depois do encarceramento; o cotidiano prisional, a dificuldade de assistência médico-psiquiátrica e a ausência de contato familiar foram apontados como fatores relacionados ao uso^13^. Neste estudo, a repetição das prescrições e a insuficiência da escuta aparecem nos casos analisados e são interpretadas à luz dessas evidências.

No que se refere ao envelhecimento em privação de liberdade, os resultados convergem com estudos nacionais que apontam o crescimento da população idosa encarcerada e a inadequação das unidades prisionais às necessidades de saúde dessa população^23^. Entre idosos encarcerados, o envelhecimento também foi associado a perdas, limitações e sofrimento intensificados pelas condições do confinamento^24^. Neste estudo, a espera prolongada, a interrupção de tratamentos e a ausência de acessibilidade foram identificadas nas vinhetas analisadas e interpretadas como manifestações de abandono assistencial.

O perfil social observado entre as pessoas privadas de liberdade confirma a função estrutural do encarceramento em massa como engrenagem do racismo institucional. Dados nacionais indicam que o sistema penal brasileiro atua como mecanismo privilegiado de gestão da pobreza e da população negra, aprofundando desigualdades previamente produzidas no acesso à educação, ao trabalho e aos serviços de saúde^4,10,25^.

No campo das identidades dissidentes, os resultados dialogam com estudos sobre as violações enfrentadas por travestis e mulheres transexuais no sistema prisional. O desrespeito ao nome social, o impedimento da hormonização e o encarceramento em instituições masculinas comprometem o reconhecimento da identidade e o acesso ao cuidado^15^. Narrativas de travestis e mulheres transexuais encarceradas também mostram dificuldades institucionais para lidar com corpos e identidades transfemininas, além da continuidade de violações durante o cumprimento da pena^26^.

A tensão entre a lógica securitária e a ética do cuidado emerge neste estudo como eixo estruturante das práticas institucionais analisadas. A PNAISP estabelece diretrizes de integralidade, intersetorialidade, descentralização, hierarquização e humanização^27^. No plano institucional, a execução das políticas penais envolve desafios de gestão, articulação e implementação^28^. Entretanto, o estudo realizado em unidades prisionais da Paraíba identificou carências estruturais, desarticulação entre os setores da saúde e da justiça e fragilidades na formação profissional^19^.

Ao mesmo tempo, os resultados evidenciam a presença de práticas cotidianas de cuidado e solidariedade que tensionam, sem romantização, a lógica colonial e necropolítica dominante. Oficinas, grupos de memória, redes informais de apoio ao tratamento, práticas de solidariedade material e expressões de espiritualidade não devem ser compreendidas automaticamente como insurgência, mas como zonas de fricção nas quais práticas institucionais, apropriações coletivas e dinâmicas micropolíticas de resistência se entrelaçam^20,21,29,30^.

Do ponto de vista teórico-analítico, os achados confirmam a pertinência de articular colonialidade de gênero, necropolítica e micropolítica do cuidado para compreender o funcionamento do cárcere brasileiro. A medicalização, a omissão assistencial e o apagamento identitário configuram componentes estruturais de um regime de governo que seleciona quais vidas devem ser protegidas e quais podem ser progressivamente eliminadas^1,2,14,22^. Simultaneamente, as brechas micropolíticas e os rearranjos cotidianos do cuidado observados indicam que a vida nunca é completamente capturada pela lógica colonial, produzindo vínculos, narrativas e formas alternativas de existir^29^.

Para o campo da Saúde Coletiva, este estudo aponta para a necessidade de aprofundar a análise crítica da violência institucional e do abandono assistencial no sistema prisional. Ao mesmo tempo, experiências de Educação Permanente em Saúde nesse contexto mostram que rodas de conversa podem favorecer discussões aprofundadas, construções coletivas e propostas de intervenção^31^. Com base nos resultados deste estudo, essas práticas de cuidado são interpretadas como uma dimensão ética e política de enfrentamento da necropolítica^14,32,33^.

## CONSIDERAÇÕES FINAIS

Este estudo analisou o sistema prisional brasileiro como território privilegiado de atualização contemporânea da colonialidade de gênero e da necropolítica, evidenciando como práticas institucionais de medicalização, omissão assistencial, apagamento identitário e seletividade racial operam de forma articulada na produção de vidas consideradas descartáveis. Os resultados demonstram que o cárcere não se limita à execução da pena, mas atua como dispositivo ativo de governo dos corpos, no qual a gestão da dor, da espera e da invisibilidade constitui tecnologia central de punição.

Ao mesmo tempo, o estudo evidenciou que, mesmo em contextos de extrema violência institucional, emergem práticas institucionais e coletivas de cuidado que tensionam a lógica colonial dominante. Oficinas terapêuticas, grupos de memória, redes solidárias de apoio ao tratamento, práticas espirituais e gestos cotidianos de reconhecimento configuram respostas concretas à ausência estatal, operando como zonas de fricção no dispositivo necropolítico e afirmando a dignidade onde a morte lenta tende a ser naturalizada. Essas práticas não devem ser romantizadas como exterioridade ao dispositivo: variam entre ofertas institucionalizadas e apropriações coletivas situadas, mas, ainda assim, compõem repertórios recorrentes de produção de cuidado e de sentido no interior do cárcere.

No campo da Saúde Coletiva, há a necessidade de reposicionar a saúde prisional como dimensão estratégica no enfrentamento das desigualdades estruturais que atravessam o sistema penal brasileiro.

O cárcere atualiza a colonialidade de gênero e a necropolítica, enquanto práticas de cuidado produzem fissuras ético-políticas que afirmam dignidade sob custódia estatal.

Embora a PNAISP represente um avanço normativo relevante, sua implementação permanece insuficiente para interromper os mecanismos de abandono assistencial, medicalização excessiva e apagamento identitário observados neste estudo. Garantir o direito à saúde nesse contexto exige não apenas ampliar a cobertura assistencial, mas também reorientar o modelo de cuidado, incorporando práticas baseadas em escuta, vínculo, reconhecimento e corresponsabilização.

Do ponto de vista ético-político, pensar o cuidado no cárcere implica questionar a centralidade do encarceramento em massa como política de gestão da pobreza, da raça, do gênero e das dissidências. Em uma sociedade marcada pela herança escravocrata e pelo racismo estrutural, a naturalização da morte lenta de mulheres, pessoas idosas, pessoas trans e jovens negros revela a persistência da colonialidade como princípio organizador das políticas de punição e controle social.

O cuidado, quando praticado como escuta qualificada, vínculo e reconhecimento, constitui uma estratégia concreta de enfrentamento à necropolítica no cotidiano prisional. Para além da mitigação de sofrimentos individuais, essas práticas reinscrevem corpos no campo da humanidade e produzem alternativas possíveis à lógica punitiva dominante. Reconhecer, fortalecer e institucionalizar tais experiências constitui tarefa central para uma saúde coletiva comprometida com a dignidade humana, a justiça social e a transformação crítica dos regimes contemporâneos de punição.
